# Bioglues Based on an Elastin-Like Recombinamer: Effect of Tannic Acid as an Additive on Tissue Adhesion and Cytocompatibility

**DOI:** 10.3390/ijms24076776

**Published:** 2023-04-05

**Authors:** Alp Sarisoy, Sergio Acosta, José Carlos Rodríguez-Cabello, Phillip Czichowski, Alexander Kopp, Stefan Jockenhoevel, Alicia Fernández-Colino

**Affiliations:** 1 Department of Biohybrid & Medical Textiles (BioTex), AME–Institute of Applied Medical Engineering, Helmholtz Institute, RWTH Aachen University, D-52074 Aachen, Germany; 2 Bioforge Lab, Group for Advanced Materials and Nanobiotechnology, Biomedical Networking Research Center of Bioengineering, Biomaterials and Nanomedicine (CIBER-BBN), Edificio LUCIA, Universidad de Valladolid, 47011 Valladolid, Spain; 3 Fibrothelium GmbH, D-52068 Aachen, Germany; 4 AMIBM-Aachen-Maastricht-Institute for Biobased Materials, Faculty of Science and Engineering, Brightlands Chemelot Campus, Maastricht University, 6167 RD Geleen, The Netherlands

**Keywords:** tissue adhesive, protein polymer, elastin-like recombinamer, tannic acid, silk fibroin, bone, skin

## Abstract

More than 260 million surgical procedures are performed worldwide each year. Although sutures and staples are widely used to reconnect tissues, they can cause further damage and increase the risk of infection. Bioadhesives have been proposed as an alternative to reconnect tissues. However, clinical adhesives that combine strong adhesion with cytocompatibility have yet to be developed. In this study, we explored the production of adhesives based on protein-engineered polymers bioinspired by the sequence of elastin (i.e., elastin-like recombinamers, ELRs). We hypothesized that the combination of polyphenols (i.e., tannic acid, TA) and ELRs would produce an adhesive coacervate (ELR+TA), as reported for other protein polymers such as silk fibroin (SF). Notably, the adhesion of ELR alone surpassed that of ELR+TA. Indeed, ELR alone achieved adhesive strengths of 88.8 ± 33.2 kPa and 17.0 ± 2.0 kPa on porcine bone and skin tissues, respectively. This surprising result led us to explore a multicomponent bioadhesive to encompass the complementary roles of elastin (mimicked here by ELR) and silk fibroin (SF), and subsequently mirror more closely the multicomponent nature of the extracellular matrix. Tensile testing showed that ELR+SF achieved an adhesive strength of 123.3 ± 60.2 kPa on porcine bone and excellent cytocompatibility. To express this in a more visual and intuitive way, a small surface of only 2.5 cm^2^ was able to lift at least 2 kg of weight. This opens the door for further studies focusing on the ability of protein-engineered polymers to adhere to biological tissues without further chemical modification for applications in tissue engineering.

## 1. Introduction

Conventional approaches to seal tissue incisions have major limitations. Sutures are usually time-consuming and technically challenging. Alternatively, staples benefit from easier placement, but they can increase the risk of infection [1]. Additionally, these procedures are suboptimal for many clinical applications, especially for sealing hard-to-reach areas or preventing fluid leakage from the incision. In this regard, tissue adhesives and sealants have emerged as an alternative to reconnect and repair ruptured tissues [2]. They seal without further damage to the tissue and are easier to apply to diverse tissues (i.e., bone, skin, cartilage, or cardiovascular tissue) [3,4]. However, commercially available adhesives have some limitations. For example, fibrin-based bioadhesives show limited adhesion to wet surfaces and there is a risk of disease transmission due to their human or animal origin. On the other hand, cyanoacrylate-derived adhesives break down into toxic by-products [5,6,7]. Further improvements in sealant materials are therefore needed to combine high adhesive strength with excellent biocompatibility.

In an attempt to develop safe and strong sealants, biomimetic approaches have been increasingly investigated in recent years. Among them are those based on plant-based polyphenols that contain catechol and gallol groups [8]. Tannic acid (TA), a natural polyphenolic compound, is abundant in plants [9]. It has attracted significant interest in the biomedical field due to its anticancer, antibacterial, and anti-inflammatory properties. Furthermore, its polyphenolic structure composed of gallol groups allows it to bind and cross-link proteins via hydrogen bonds, electrostatic, and hydrophobic interactions [10]. TA, therefore, achieves water-resistant adhesion [11,12] and has hemostatic properties due to its ability to bind blood cells and components [13]. For the development of tissue adhesives, TA has been combined with synthetic and natural polymers. For example, TA has been used as a primer on skin tissue together with thermally responsive polymers (e.g., PNIPAM copolymers) as a cohesive matrix to obtain a reversible adhesive [14]. TA has also been combined with silk fibroin (SF) due to its high mechanical strength and cytocompatibility, resulting in water-immiscible tissue adhesives [15,16]. However, synthetic and also some natural polymers have certain drawbacks, such as immunogenicity or a lack of bioactivity, among others [17]. These limitations can be overcome by protein-engineered polymers that combine the best of both the biological and technical worlds: bioactivity and customization.

Elastin-like recombinamers (ELRs) are protein-engineered polymers bioinspired by elastin in the extracellular matrix (ECM) [18]. They are based on the repetition of hydrophobic sequences present in tropoelastin, typically the pentapeptide Val-Pro-Gly-Xaa-Gly. This confers valuable physicochemical and biomechanical properties for use in tissue engineering. ELRs exhibit lower critical solution temperature (LCST) phase behavior in aqueous solution. At a certain temperature (the so-called transition temperature (*T**_t_***) or LCST cloud point), ELRs undergo coacervation, driven by an increase in the number of β-turn structures [19]. This phase transition offers the ability to create defined molecular architectures by tuning the ELR sequence [20,21]. In addition, ELRs can be custom-made to create 3D cell-instructive scaffolds with multiple functionalities, such as integrin-binding or metalloprotease-sensitive sequences. Due to their inherent elasticity and resilience, they are excellent candidates for promoting in situ regeneration of elastic tissues, such as vascular tissues and skin [22,23], but can also be engineered and processed to meet demands for the regeneration of tissues with very different properties and compositions, including neural tissue, cartilage, or bone [24]. All of these properties have led to the exploration of the use of ELRs as bioadhesives [25,26,27], mainly in combination with catechol groups, which are incorporated into the ELR chain either enzymatically [28] or chemically [27]. However, the use of catechol groups as adhesive molecules requires careful control of the oxidation state, and toxic chemicals such as sodium periodate may be necessary [29,30,31]. 

Here we aimed to develop a tissue adhesive based on a protein-engineered polymer without the need for chemical modification. First, we investigated the effect of combining TA-based glue with an ELR for the development of biomimetic and biocompatible sealants. We evaluated the adhesive properties of the resulting mixtures in two models of dry (bone) and wet (skin) tissues as well as their cytotoxicity against primary human cells. Unlike previously described TA-containing adhesives, we found that TA, besides compromising cytocompatibility, has a counter-productive effect when combined with ELRs. Notably, ELR, either alone or combined with SF, showed excellent adhesion (~88 and 123 kPa, respectively, in bone tissue) and cytocompatibility.

## 2. Results and Discussion

### 2.1. Preparation and Optimization of ELR-Based Bioadhesives

**Figure 1:**
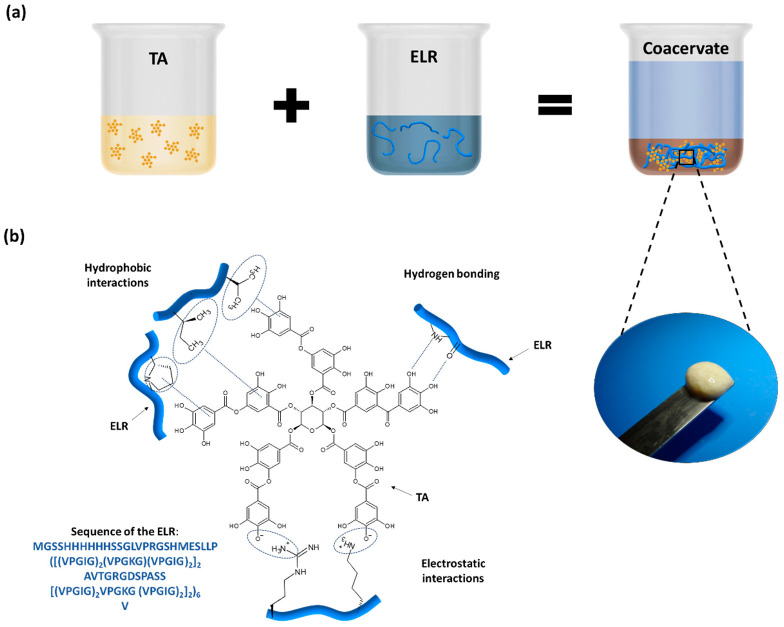
(**a**) Preparation of ELR + TA bioadhesive and coacervate formation. (**b**) Schematic of the potential interactions between ELR and TA.

The preparation of the ELR + TA bioadhesive is schematically shown in Figure 1a. In order to prevent the phase transition and folding of the ELR and thus maximize the interaction between the TA molecules and ELR backbone, both solutions were pre-chilled before mixing. Instantaneously after mixing, we observed the formation of a coacervate (Figure 1a). This phenomenon confirmed the capability of TA to act as a physical crosslinker of proteins via interactions between gallol groups and proline [32,33] and the formation of multiple non-covalent interactions, such as hydrogen bonds as well as hydrophobic and electrostatic interactions (Figure 1b) [8,10].

**Figure 2:**
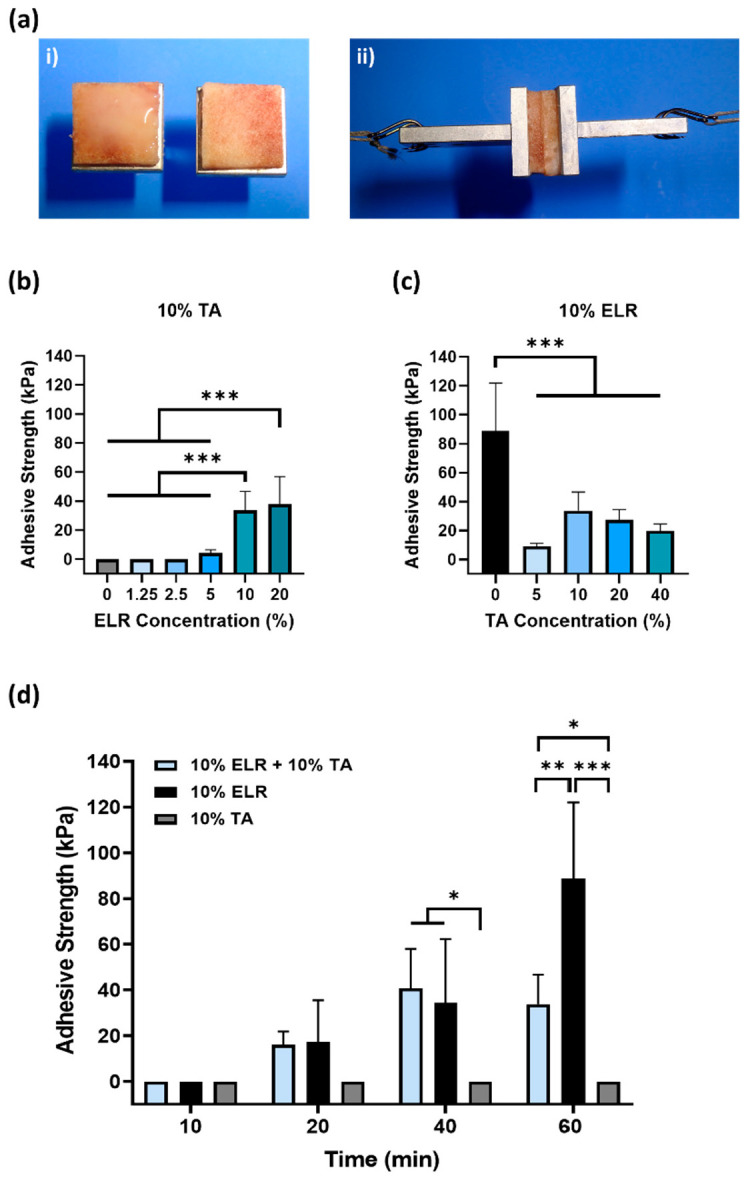
Evaluation of the adhesive properties of ELR-based bioadhesives on bone tissue. (**ai**) Image of the bone tissue bonded to specimen holders; the ELR-based bioadhesive is applied to the bone surface and, subsequently, both bone surfaces are placed in contact with each other. (**aii**) Image of the specimen during tensile testing. (**b**) Influence of the ELR concentration on adhesive strength. (**c**) Influence of the TA concentration on adhesive strength. (**d**) Effect of curing time (10, 20, 40, and 60 min) on the adhesive strength (* *p* < 0.05, ** *p* < 0.01, *** *p* < 0.001). All the data in (**b**–**d**) are shown in the Supplementary Materials (Tables S1–S3).

To check whether the ELR + TA biohybrid coacervate features adhesive properties, we performed adhesion tests according to ASTM, as described above [34]. We used cancellous porcine bone (Figure 2a) as a model tissue of trabecular bone in the mandible [35], given the potential of ELRs for dental applications [36,37,38]. 

First, we evaluated the effect of the ELR concentration on adhesion (Figure 2b). Different ELR + TA blends were prepared with increasing concentrations of ELR (0–20%), maintaining a constant concentration of 10% TA. After tensile testing, we found that only the 10% TA solution (without ELRs) could not withstand any load. Although TA can bind to multiple tissues [39], it must be combined with a polymer that provides cohesion to the adhesive [14,15,16,40,41]. The lowest concentrations of ELR (i.e., 1.25% and 2.5%) did not achieve adhesion to the bone surface, but increasing the ELR concentration in the ELR + TA blends led to bioadhesive coacervates. The adhesion increased significantly (*** *p* < 0.001) from 4.4 ± 2.0 kPa (5% ELR) to 33.8 ± 12.9 kPa (10% ELR) and 38.1 ± 18.9 kPa (20% ELR). This shows that it is necessary to add concentrations of ELR equal to or higher than 5% for the mixture to result in an adhesive coacervate.

Next, we tested different ELR+TA formulations with increasing concentrations of TA. As shown in Figure 2c, the adhesive strength increased from 9.0 ± 2.1 kPa for the coacervate with 5% TA to 33.8 ± 12.9 kPa with 10% TA. At higher TA concentrations, the adhesive strength of the bioadhesive showed a decreasing trend, with values of 27.4 ± 7.1 kPa and 20.0 ± 4.5 kPa for the ELR + TA coacervates with 20 and 40% TA, respectively. This trend is consistent with a previous study [16]. The excess of TA may result in the formation of secondary TA networks that could affect the cohesion forces in the adhesive coacervate. Surprisingly, the 10% ELR solution (without TA) had a higher adhesive strength (88.8 ± 33.2 kPa, *** *p* < 0.001) than any of the ELR + TA mixtures. This may reflect a combination of two driving forces: adhesive and cohesive forces. On one hand, the ELR chains could adhere to the tissue surface through the formation of non-covalent interactions, mainly hydrogen bonds involving the hydroxyl groups of Ser (6%) and Thr (1.5%), and electrostatic interactions involving the positively charged side chains of Lys (3%), Arg (1.8%), and His (1.8%). These interactions at the molecular level could be complemented by the porous topography of the trabecular bone, facilitating the interpenetration of the ELR chains to form physical entanglements on the surface. On the other hand, cohesiveness could reflect the physical crosslinking of the ELR chains. ELRs undergo thermally-driven LCST phase behavior, and above a certain temperature (*T**_t_***), they self-assemble into insoluble coacervates [21,42]. In this case, the ELR used has a *T**_t_*** of ~21 °C [43], so that at 37 °C, the ELR chains are folded and stabilized by hydrophobic interactions that could impart cohesive forces within the adhesive. 

**Figure 3:**
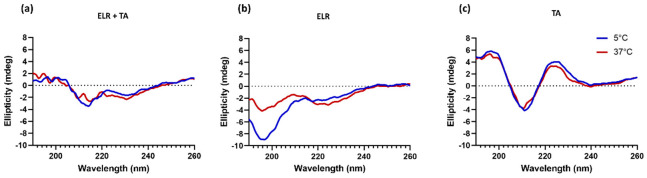
Secondary structure characterization by circular dichroism spectroscopy of (**a**) ELR + TA, (**b**) ELR, and (**c**) TA solutions.

It was striking that the adhesive strength decreased when ELR was combined with TA compared to ELR alone. This indicates that the interaction between TA molecules and ELRs might have a counterproductive effect. We hypothesize that this phenomenon is a combination of two effects. First, the complexing and crosslinking of TA molecules through hydrogen bonds, as well as electrostatic and hydrophobic interactions, could limit the number of groups available in both TA and ELR to bind to the tissue surface. In fact, all ELR + TA formulations showed an adhesion failure on porcine bone after the tensile testing (Table S4, Supplementary Materials), indicating that the interaction at the interface between the bone and the tissue adhesive is the weak point for these formulations. Another effect could be the high affinity of TA for proline residues [32,33], one of the key amino acids of the characteristic pentapeptide monomer of ELRs (Val-Pro-Gly-Xaa-Gly). Proline plays a key role in the phase transition of ELRs and the intrinsic disorder of ELRs [42]. TA complexation with ELRs seems to interfere with the self-assembly of ELRs. To gain further insight into this point, the conformational state of ELR in the presence of TA was analyzed by circular dichroism in parallel to that of ELR alone (Figure 3). The ELR solution spectra show the characteristic peak of intrinsically disordered proteins (~197 nm) below *T**_t_***, at 5 °C [44]. As the temperature increased to above *T**_t_***, this peak decreases and a shoulder appears around 210. This signal is characteristic of a higher proportion of type II β-turns and distorted β-sheet conformations [45,46], thus demonstrating the thermoresponsive behavior of ELR alone, as demonstrated for other ELRs [21,47]. In contrast, the spectra of ELR + TA at 5 °C are almost identical to those at 37 °C, speaking to the interference of TA on the typical ELR self-assembly. TA promotes ELR coacervation below the *T**_t_*** and at the same time deprives ELR of its elastic behavior. Indeed, the ELR + TA coacervate was rather stiff, lacking the intrinsic elasticity of elastin-like recombinamers. For further studies, 10% ELR and 10% ELR + 10% TA adhesives were selected as the formulations with optimal adhesive strength.

In order to gain further insight into the performance of ELR alone and ELR+TA, the adhesion strength was investigated at shorter time intervals (Figure 2d), as adhesive properties after rather short incubation periods are highly relevant for biomedical applications, yet are very challenging to achieve. Specifically, the adhesiveness of the ELR solution (10% ELR), TA solution (10% TA), and ELR + TA coacervate (10% ELR + 10% TA) was studied after incubation of the samples for 10, 20, and 40 min at 37 °C. We observed that both the ELR solution and the ELR+TA coacervate showed adhesive effects after incubation for only 20 min, with adhesion values of 17.2 ± 18.3 kPa and 16.0 ± 5.9 kPa, respectively. In both formulations, the adhesive properties increased over time. After incubation for 40 min, both experienced an almost equal increase, reaching values of 40.7 ± 17.3 kPa (ELR + TA) and 34.5 ± 27.8 kPa (ELR). However, whereas incubation for 60 min did not cause any apparent change in the adhesive strength of the ELR+TA coacervate to the bone tissue, the adhesive strength of ELR increased more than twofold (88.8 ± 33.2 kPa, *** *p* < 0.001). This increase in adhesive strength may be associated with the progressive evaporation of water molecules from both the ELR–tissue interface and the interior of the ELR network, which would increase interactions with the tissue surface and increase the packing of the ELR chains.

### 2.2. Adhesiveness to Wet Tissues

To gain more insight into the adhesiveness of ELR-based bioadhesives, we also tested them on porcine skin as an alternative surface. This was chosen as a model of wet tissue because it closely resembles human skin [48] and is commonly used to test bioadhesives [34]. 

**Figure 4:**
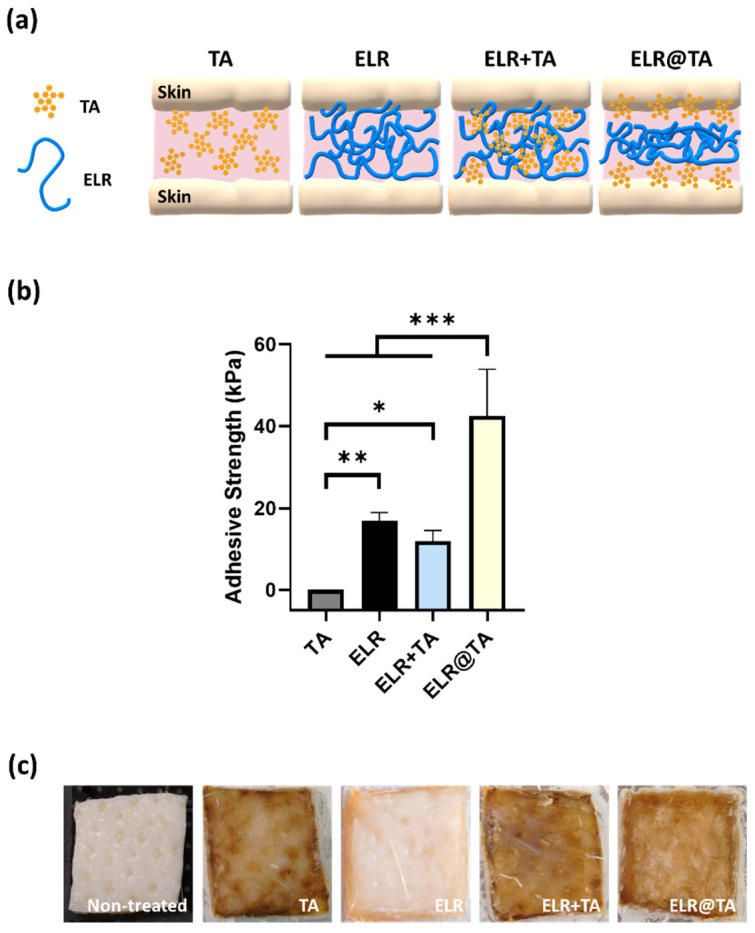
Evaluation of the adhesiveness of ELR-based bioadhesives on skin tissues. (**a**) Schematic representation of ELR-based bioadhesives on skin tissue. (**b**) Adhesive strength on porcine skin of the different formulations (10% TA, 10% ELR, 10% ELR + 10% TA, and 10% ELR @ 10% TA) (* *p* < 0.05, ** *p* < 0.01, *** *p* < 0.001). (**c**) Photographs of the skin before and after testing with the different formulations.

The tensile testing test showed that the application of ELR alone or ELR + TA coacervate resulted in adhesive strength values of 17.0 ± 2.0 kPa and 12.0 ± 2.6 kPa, respectively (Figure 4b), while only TA (control) on the skin tissue was insufficient to achieve an adhesive effect. The adhesive strength values were significantly lower on the skin than on bone tissue, which may be due to the different compositions and topography of the tissues. However, similar to the bone adhesion testing, the application of the ELR solution alone provided better adhesion than the ELR + TA coacervate.

Because all formulations based on the ELR+TA coacervate resulted in lower values than the ELR solution, we tested an additional formulation (ELR@TA, schematically represented in Figure 4a). This formulation is based on the sequential application of both components (i.e., TA as a molecular bioglue and ELR as a cohesive polymer matrix) and has shown promising results in previous studies with natural and synthetic thermoresponsive polymers [14]. In detail, the ELR@TA biohybrid adhesive was applied in two steps to prevent ELR phase separation before coming into contact with the tissue. A TA solution (10% *w*/*v*) was first applied as a priming layer on the surface of the tissue and then an ELR solution (10% *w*/*v*) was applied as a dissipative matrix (ELR@TA, Figure 4a). Therefore, both the adhesion of TA to the tissue and the cohesive interactions between the ELR chains can be maximized. Indeed, the deposition of the TA priming layer resulted in a more than twofold improvement (*** *p* < 0.001) in its adhesive strength on skin tissue, achieving values of 42.5 ± 11.4 kPa (Figure 4b). However, the macroscopic evaluation of skin tissues after tensile testing revealed that the surface of the tissue was altered when TA was applied (Figure 4c). All tissues treated with TA or in contact with an ELR + TA formulation turned brown, whereas the ELR application did not affect the macroscopic properties (i.e., tissue coloration). This effect may be due to the high binding affinity of TA to biological tissues. It can fix biological membranes and cell glycocalyx, but it also complexes proteins [49,50,51]. These effects are of great interest for their application as adhesives, but their impact on cell viability must also be taken into account.

### 2.3. Cytocompatibility

Given that TA altered the skin surface (Figure 4c), we evaluated the cytocompatibility of the adhesive formulations to check whether these alterations were associated with a cytotoxic effect. We assessed the viability of primary human SMCs using an indirect test according to the ISO-10993-12:2019 standard [52].

**Figure 5:**
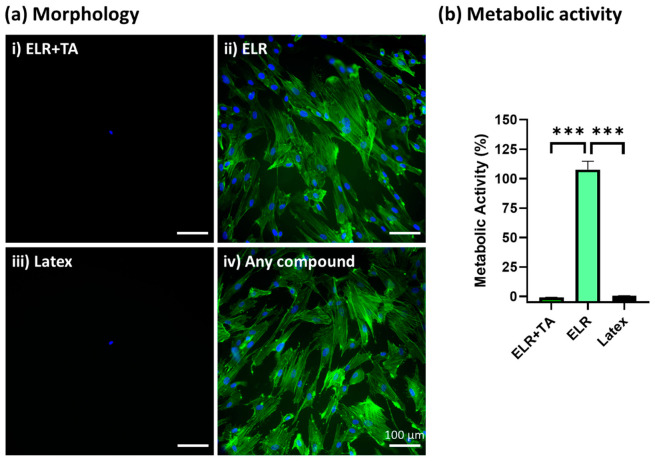
In vitro cytocompatibility assessment of ELR-based bioadhesives. (**a**) Fluorescence microscopy images of primary human SMCs cultured with (**i**) extracted media from 10% ELR + 10% TA, (**ii**) extracted media from 10% ELR, (**iii**) extracted media from latex (positive control), and (**iv**) DMEM (negative control). The actin filaments of the cytoskeleton and nuclei are labeled with phalloidin-488 (green) and DAPI (blue), respectively. (**b**) Metabolic activity of primary human SMCs incubated with extracted media from the adhesives (*** *p* < 0.001).

After the incubation of SMCs with extracted media from the different formulations for 24 h, ELR + TA showed high cytotoxicity and no cells were observed under the fluorescence microscope (Figure 5(ai)), similar to the positive control (Figure 5(aiii), latex). In contrast, ELR alone achieved excellent cytocompatibility, with the cells showing a confluent and well-spread morphology (Figure 5(aii)). In addition to a morphological evaluation, a metabolic test was carried out (Figure 5b), which confirmed that the ELR + TA formulation was cytotoxic. No metabolic activity was detected, as was the case for the positive control (latex, 0% viability). These results were striking considering the increasing number of studies exploring TA in the context of biomedical applications. For example, TA has previously been explored for the development of hemostatic sealants, and typically low or no cytotoxic activity has been reported [15,16]. However, in addition to its ability to fix several macromolecules and drive protein coacervation [49,50,51], it can behave as a pro-oxidant or antioxidant molecule depending on the concentration [53,54]. TA can induce the production of reactive oxygen species (ROS) by interacting with copper ions in chromatin, thus damaging DNA [55]. This may be one of the causes of the observed cytotoxicity at high concentrations [56]. The release of TA from the bioadhesive will be studied in depth in the future because this could be beneficial with controlled delivery dynamics. In the case of ELRs, metabolic activity reached more than 100% (105.9 ± 5.8%), which is consistent with previous studies [57].

### 2.4. Preparation of Protein Composite Bioadhesive Based on ELR and SF

The encouraging adhesiveness and cytocompatibility results for the ELR-based formulations (without TA) led us to hypothesize the possibility to develop a bioadhesive that mimics, even more closely, the multi-component nature of the ECM, while still achieving outstanding adhesive properties. Because ELRs are intrinsically disordered polypeptides characterized by stimuli-responsive self-assembling behavior and elastic mechanical properties (i.e., low stiffness, high extensibility, and resilience) [18], we attempted to combine them with another protein material with complementary properties, SF. SF is widely used for tissue engineering applications due to its cytocompatibility and mechanical properties [58,59]. Its sequence shows a high degree of repeatability, but it folds into crystalline structures with a high proportion of β-sheets [60]. These highly ordered networks lead to great strength and stiffness that could be synergistically combined with the elastic ELR to increase mechanical strength and cohesiveness within the bioadhesive.

**Figure 6:**
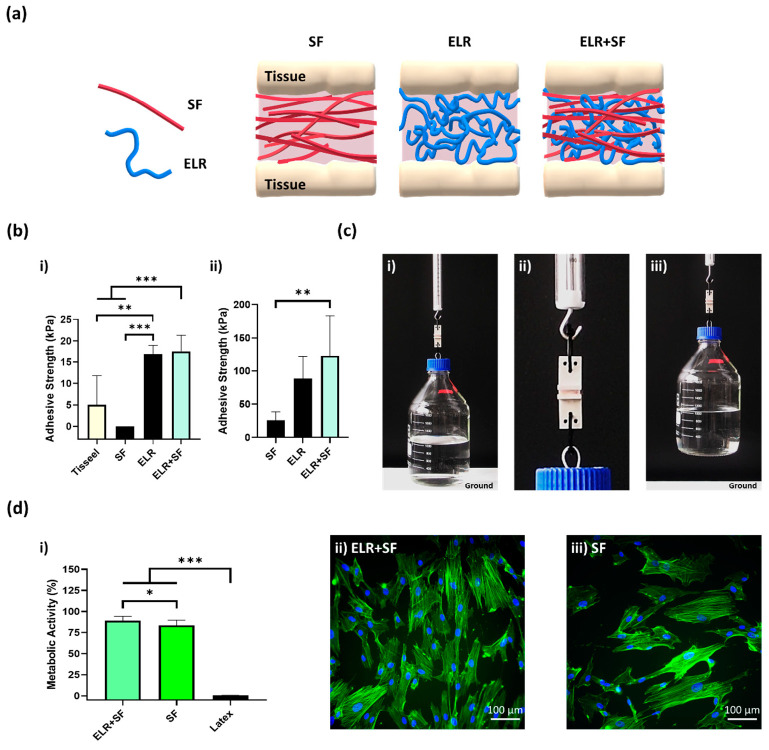
(**a**) Schematic representation of a composite bioadhesive based on SF and ELR. (**b**) The adhesive strength of the composite bioadhesive (10% ELR + 10% SF) on (**bi**) porcine skin and (**bii**) bone tissue. (**c**) Lifting of a glass bottle containing 1 L of water (overall weight 2 kg) with 10% ELR + 10% SF with 2.5 cm^2^ of porcine bone. (**ci**) Image just before lifting, (**cii**) close-up image of the bone surfaces glued with the bioadhesive 10% ELR + 10% SF, and (**ciii**) 2-kg-weight bottle suspended in the air. (**di**) Metabolic activity of SMCs incubated with the extracted media of the adhesives (* *p* < 0.05, ** *p* < 0.01, *** *p* < 0.001). Morphological images of SMCs under a fluorescence microscope after incubation with extracted media from (**dii**) ELR + SF and (**diii**) SF. The actin filaments of the cytoskeleton and nuclei are labeled with phalloidin-488 (green) and DAPI (blue), respectively.

The protein composite bioadhesive was prepared by simply mixing equal volumes of SF and ELR to obtain a final concentration of 10% ELR and 10% SF. The appearance of the ELR + SF bioadhesive is shown in Figure S1 (Supplementary Materials). We then applied the protein composite solutions to the skin and bone tissues (schematically represented in Figure 6a) and tested the tensile strength. Although the 10% SF solution gave null adhesion values on porcine skin (Figure 6(bi)), the composite bioadhesive (ELR + SF) matched the adhesive properties of ELR and achieved an adhesive strength approximately three times higher (17.6 ± 3.7 kPa, *** *p* < 0.001) than Tisseel, the commercially available fibrin glue (5.0 ± 6.8 kPa).

In the bone tissue (Figure 6(bii)), the adhesion strength was one order of magnitude greater than on the skin for all formulations. This may reflect the different properties of the tissues and the drying of the protein adhesive that could promote the formation of physical entanglements of the protein polymers on the tissue surface. It was remarkable that the ELR and ELR + SF formulations achieved adhesiveness values of 88.8 ± 33.2 kPa and 123.3 ± 60.2 kPa, respectively. Thus, the composite formulation improved the adhesiveness of the bioadhesive. This may be due to the additive effect of two factors. SF has the ability to self-assemble into highly stable beta-like structures, which will contribute to increasing the cohesive forces within the ELR-based adhesive; however, in addition, SF has a high content of amino acids, with groups capable of forming hydrogen bonds with the tissue surface, such as hydroxyl groups (Ser (~12%) and Tyr (~5.5%)) and carboxyl groups (Asp and Glu (~1.5%)), which appear to contribute to increasing the adhesive properties of the bioadhesive. For a better demonstration of the adhesion power of this formulation, we applied it to a 2.5 cm^2^ bone tissue surface and we were able to lift 2 kg (Figure 6(ci–iii)). Additionally, good metabolic activity (90.4% ± 6.7, Figure 6(di)) and well-spread morphology (Figure 6(dii)) were observed with the ELR + SF formulation. The bioadhesive ELR + SF shows not only adhesive properties comparable to other very promising adhesives (Table S5, Supplementary Materials), but it does so with a very simple formulation, avoiding the use of any harmful components that could otherwise compromise the biomedical application. The composite bioadhesive showed encouraging results regarding adhesiveness and cell viability, which sets the basis for the future and necessary validation of the bioadhesives in in vivo settings.

## 3. Materials and Methods

### 3.1. Preparation of Bioadhesives

ELR solutions were prepared by dissolving lyophilized ELR (azide-functionalized, RGD-bearing ELR; TP20254, Technical Proteins Nanobiotechnology, Valladolid, Spain) in cold Arium Pro ultrapure water (Sartorius, Göttingen, Germany) at concentrations of 2.5%, 5%, 10%, 20%, and 40% (*w*/*v*). The ELR solutions were then incubated at 4 °C overnight to ensure the complete dissolution of the ELR. 

TA solutions were prepared by dissolving TA (Sigma-Aldrich, St. Louis, MO, USA) in ultrapure water at concentrations of 10%, 20%, 40%, and 80% (*w*/*v*), and stirring at room temperature (RT) until they reached a homogeneous consistency. The bioadhesive ELR + TA was prepared by mixing equal volumes of TA (pH 2.2–2.7) and ELR (pH ~7) solutions at 4 °C. After mixing for 30 s, the resulting coacervate was used as an adhesive. Additionally, 10% TA was used as a priming solution to treat the skin surfaces. The tissues were primed by immersion in 10% TA for 2 min and rinsed with ultrapure water for 5 s.

SF solution (32% (*w*/*w*)) was diluted to 20% (*w*/*w*) in ultrapure water. The silk fibroin aqueous solution was obtained using PureSilk technology (Fibrothelium, Aachen, Germany) enabling medical-grade quality on an industrial scale for a broad range of concentrations. Briefly, SF was separated from sericin by degumming it in a hot alkali solution before dissolving it in a proprietary non-toxic solvent system based on Ajisawa’s reagent. The dissolved fibroin was fully dialyzed against VE water for 8 h by tailored extraction processing. Two different stock SF solutions (pH 8.16 and 8.3) were used for the experiments. Equal volumes of 20% SF and 20% ELR were mixed at 4 °C to make the final concentration of 10% ELR + 10% SF.

As a reference, commercially available fibrin sealant purchased from Tisseel (Baxter International, Deerfield, IL, USA) was prepared as recommended by the manufacturer. 

### 3.2. Evaluation of Adhesive Properties

Adhesive strength was measured on porcine bone and skin tissues (samples of approximately 1.5 cm^2^) using a modified ASTM–F2258–05 standard test [34]. Trabecular bone samples were prepared from porcine femoral heads obtained from a local butcher using a cut-grinder primus 2 diamond pathology saw (Biosystems Switzerland, Basel, Switzerland). Porcine skin samples were prepared from porcine tissue provided by the Animal Facility of the University Hospital Aachen (Aachen, Germany). Briefly, the hairs attached to the skin were removed by shaving, and the subcutaneous tissue was removed. The skin samples were always kept between gauzes soaked in phosphate-buffered saline (PBS; Thermo Fisher Scientific, Waltham, MA, USA) to keep them moist. 

The tissue samples were glued to metal holders using a cyanoacrylate adhesive. The dermal part of the skin tissues was left for adhesion testing. Sufficient protein-based adhesive was applied to one side of the tissue without significant overflow, and the other side was placed on top by applying gentle pressure for 5 s. The excess amount of adhesive was trimmed, the samples were incubated at 37 °C in a humidified environment (95%) for 60 min, and bone samples were incubated at 37 °C with environmental humidity.

After incubation, the maximum adhesion force (F_max_) was measured using a biaxial testing machine (Zwick Roell, Ulm, Germany) in uniaxial mode with 50 N load cells. The tensile speed was set to 2 mm/min, and five samples were measured per condition. The surface area (A) was calculated from photographs taken of the samples after testing using ImageJ v1.53t (National Institutes of Health, Bethesda, MA, USA). The adhesive strength was defined by Equation (1):

(1) $$Adhesive\;strength\;(\mathrm{kPa})=\frac{Fmax\;(N)}{A\;({\mathrm{cm}}^{2})}$$

### 3.3. Circular Dichroism

ELR, TA, and ELR+TA were dissolved at 1 mg/mL in ultrapure water (5 °C) and diluted to a final concentration of 0.1 mg/mL immediately before the circular dichroism (CD) analysis. The CD measurements were performed in 1-mm path-length quartz cuvettes using a Jasco J-810 spectropolarimeter (Jasco, Easton, MD, USA) equipped with a temperature controller (Research Technical Services, University of Alicante, Spain). The signals were scanned over a range of 260–190 nm with a data pitch of 0.5 nm. All measurements were carried out in triplicate. Data were smoothed for representation using a second order smoothing (average of 5 neighbors).

### 3.4. Isolation and Culture of Human Umbilical Vein Smooth Muscle Cells 

Smooth muscle cells (SMCs) were isolated from human umbilical veins as previously described [61]. Briefly, the vein was washed with PBS and the endothelial cells were removed by adding 1 mg/mL collagenase (Sigma-Aldrich). The adventitia was removed, and the vein was cut into 1-mm rings and then bathed in Dulbecco’s modified Eagle’s medium (DMEM; Thermo Fisher Scientific) supplemented with 10% (*v*/*v*) fetal bovine serum (FBS; Thermo Fisher Scientific) and 1% (*v*/*v*) ABM antibiotics (PAN-Biotech, Aidenbach, Germany). To obtain a sufficient number of cells, cells were serially passaged with 0.05% trypsin/0.02% EDTA (PAN-Biotech) and cultured at 37 °C in a humidified environment (95%) with 5% CO_2_. The phenotype of the SMCs was verified by immunocytochemistry for the presence of α-smooth muscle actin (α-smA) and the absence of von Willebrand factor.

### 3.5. Cytocompatibility Assay 

Cell viability was evaluated using an indirect cytotoxicity test following standard ISO-10993-12:2019 [52]. Briefly, SMCs were fed with the extracted media obtained from (i) 10% ELR + 10% TA, (ii) 10% ELR, (iii) 10% ELR + 10% SF, and (iv) 10% SF. Latex (6 cm^2^/mL) and DMEM were used as positive and negative controls, respectively. 

The extracted medium was obtained by incubating the protein-based adhesives in DMEM at 100 mg/mL for 72 h (450 rpm, 37 °C). ELR + TA coacervate was washed prior to incubation with Dulbecco’s phosphate-buffered saline (DPBS; PAN-Biotech) for 5 min to remove excess TA. In parallel, SMCs were seeded (1500 cells/well) in a 96-well plate and incubated at 37 °C in a humidified environment (95%) with 5% CO_2_ for 72 h. The extracted medium was collected, centrifuged at 1000× *g* for 20 min, and the supernatant was used to change the medium of the SMCs. The SMCs were then incubated for 24 h (37 °C, 5% CO_2_). Cytocompatibility was then evaluated by measuring the metabolic activity and cell morphology.

#### 3.5.1. Analysis of Metabolic Activity

Metabolic activity was analyzed using the CellTiter-Blue kit (Promega, Madison, WI, USA) on an Infinite M200 plate reader (TECAN, Mannedorf, Switzerland) at 560 nm excitation and 590 nm emission. The reagent contains an indicator dye named resazurin, and viable cells can reduce resazurin to resorufin, which is highly fluorescent. Therefore, the fluorescence produced by viable cells was measured after 0.5, 1, 2, 3, and 4 h. Four hours of incubation was selected as the holding time before further analysis since the fluorescence reaches a plateau at this time. Finally, the background signal from the wells containing only the extracted medium was subtracted and the fluorescence percentage was calculated for each group by setting the fluorescence of the DMEM control to 100%.

#### 3.5.2. Morphological Analysis of SMCs

SMCs were washed with DPBS and fixed in 4% paraformaldehyde (Carl Roth, Karlsruhe, Germany) for 1 h at RT. The cells were then washed three times with DPBS and permeabilized with 0.1% Triton X-100 (Sigma-Aldrich) for 5 min. Actin filaments were stained with phalloidin-iFluor 488 conjugate (Cayman Chemicals, Ann Abor, MI, USA) in 1% (*w*/*v*) bovine serum albumin (Sigma-Aldrich) for 90 min at RT. After washing the cells three times with DPBS, the nuclei were stained with DAPI (Carl Roth) for 15 min and washed three times with DPBS. Finally, the stained SMCs were visualized under an Eclipse Ti fluorescence microscope (Nikon Instruments, Tokyo, Japan) using a 20× objective.

### 3.6. Statistical Analysis

All data are presented as the mean ± standard deviation (SD). Statistical analysis between three or more groups was performed by one-way analysis of variance (ANOVA) with the Holm–Šidák *post hoc* test. The difference between the two groups was assessed using the Student’s *t*-test. A *p*-value < 0.05 was considered statistically significant (* *p* < 0.05, ** *p* < 0.01, *** *p* < 0.001).

## 4. Conclusions

We studied the adhesion of ELR-based bioadhesives and demonstrated their ability to adhere to biological tissues. ELR-based bioadhesives showed promising adhesive strength on bone and skin tissues without the need for cathecol functionalization. The combination of ELR with TA polyphenol leads to the formation of a coacervate with adhesive properties inferior to those of ELR solutions and with cytotoxic side effects. Interestingly, we showed that the combination of the intrinsically disordered protein polymer ELR with highly ordered protein SF led to safe and strong bioadhesive composites. The ELR + SF composite gave adhesion values on skin three times higher than those achieved by a commercially available fibrin glue and could sustain a weight of 2 kg when applied to a bone surface area of 2.5 cm^2^. These features make ELRs potential candidates for their use as adhesives in future studies focusing on tissue engineering applications. In addition, the sequence of ELRs can be engineered according to clinical needs, i.e., implementing metalloprotease-sensitive sequences for controlled degradation on wound healing applications [62], which opens new horizons for tuning the bioglue’s performance.

## Data Availability

Data associated with this study are available upon request to the corresponding authors.

## Supplementary Materials

The supporting information can be downloaded at: https://www.mdpi.com/article/10.3390/ijms24076776/s1. References [63,64,65,66,67,68,69] are cited in the supplementary materials.
