# Supplementary material for: Bioglues Based on an Elastin-Like Recombinamer: Effect of Tannic Acid as an Additive on Tissue Adhesion and Cytocompatibility
Source: Int J Mol Sci. 2023 Apr 5;24(7):6776. doi: 10.3390/ijms24076776 (PMC10095112; doi:10.3390/ijms24076776)
Supplement: Supplementary file 1 [file ijms-24-06776-s001.zip › ijms-2271620-supplementary.pdf]

Supplementary Materials

Bioglues Based on an Elastin-Like Recombinamer: Effect of Tannic Acid as an Additive on Tissue Adhesion and Cytocompatibility

Alp Sarisoy [1,†], Sergio Acosta [1,†], José Carlos Rodríguez-Cabello [2], Phillip Czichowski [3], Alexander Kopp [3], Stefan Jockenhoevel [1,4,*] and Alicia Fernández-Colino [1,*]

[1] Department of Biohybrid & Medical Textiles (BioTex), AME–Institute of Applied Medical Engineering, Helmholtz Institute, RWTH Aachen University, D-52074 Aachen, Germany

[2] Bioforge Lab, Group for Advanced Materials and Nanobiotechnology, Biomedical Networking Research Center of Bioengineering, Biomaterials and Nanomedicine (CIBER-BBN), Edificio LUCIA, Universidad de Valladolid, 47011, Valladolid, Spain

[3] Fibrothelium GmbH, D-52068 Aachen, Germany

[4] AMIBM-Aachen-Maastricht-Institute for Biobased Materials, Faculty of Science and Engineering, Brightlands Chemelot Campus, Maastricht University, 6167 RD Geleen, The Netherlands

* Correspondence: jockenhoevel@ame.rwth-aachen.de (S.J.); fernandez@ame.rwth-aachen.de (A.F.-C.); Tel.: +49-241-80-47478 (S.J.); +49-241-80-47470 (A.F.-C.)

† These authors contributed equally to this work.

Table S1. Adhesive strength (expressed as mean ± SD) of different bioadhesive formulation, differing the ELR concentration, as represented in Figure 2b.

Formulation	Adhesive strength (kPa)
10% TA	0.0 ± 0.0
1.25% ELR + 10% TA	0.0 ± 0.0
2.5% ELR + 10% TA	0.0 ± 0.0
5% ELR + 10% TA	4.4 ± 2.0
10% ELR + 10% TA	33.8 ± 12.9
20% ELR + 10% TA	38.1 ± 18.9

Table S2. Adhesive strength (expressed as mean ± SD) of different bioadhesive formulation, differing the TA concentration, as represented in Figure 2c

Formulation	Adhesive strength (kPa)
10% ELR	88.8 ± 33.2
10% ELR + 5% TA	9.0 ± 2.1
10% ELR + 10% TA	33.8 ± 12.9
10% ELR + 20% TA	27.4 ± 7.1
10% ELR + 40% TA	20.0 ± 4.5

Table S3. Adhesive strength of the bioadhesive formulations along time, (10, 20, 40 and 60 min), as presented in Figure 2d.

	Time (min)			
**Formulation**	**10**	**20**	**40**	**60**
**10% ELR + 10% TA**	0.0 ± 0.0	16.0 ± 5.9	40.7 ± 17.3	33.8 ± 12.9
**10% ELR**	0.0 ± 0.0	17.1 ± 18.3	34.5 ± 27.8	88.8 ± 33.2
**10% TA**	0.0 ± 0.0	0.0 ± 0.0	0.0 ± 0.0	0.0 ± 0.0

Table S4. Failure types of the bioadhesive formulations. The images show the appearance of the porcine bone samples after adhesion tests.

Formulation	Images	Failure type
5% ELR + 10% TA	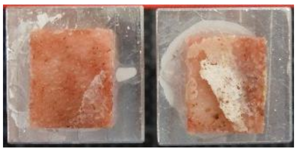	Adhesion
10% ELR + 10% TA	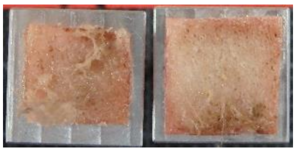	Adhesion
20% ELR + 10% TA	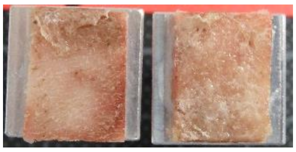	Adhesion
10% ELR + 5% TA	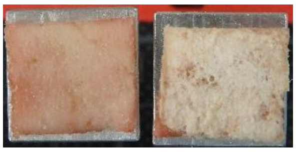	Adhesion
10% ELR + 20% TA	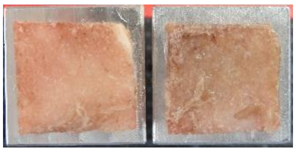	Adhesion
10% ELR + 40% TA	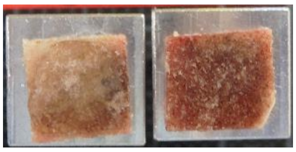	Adhesion

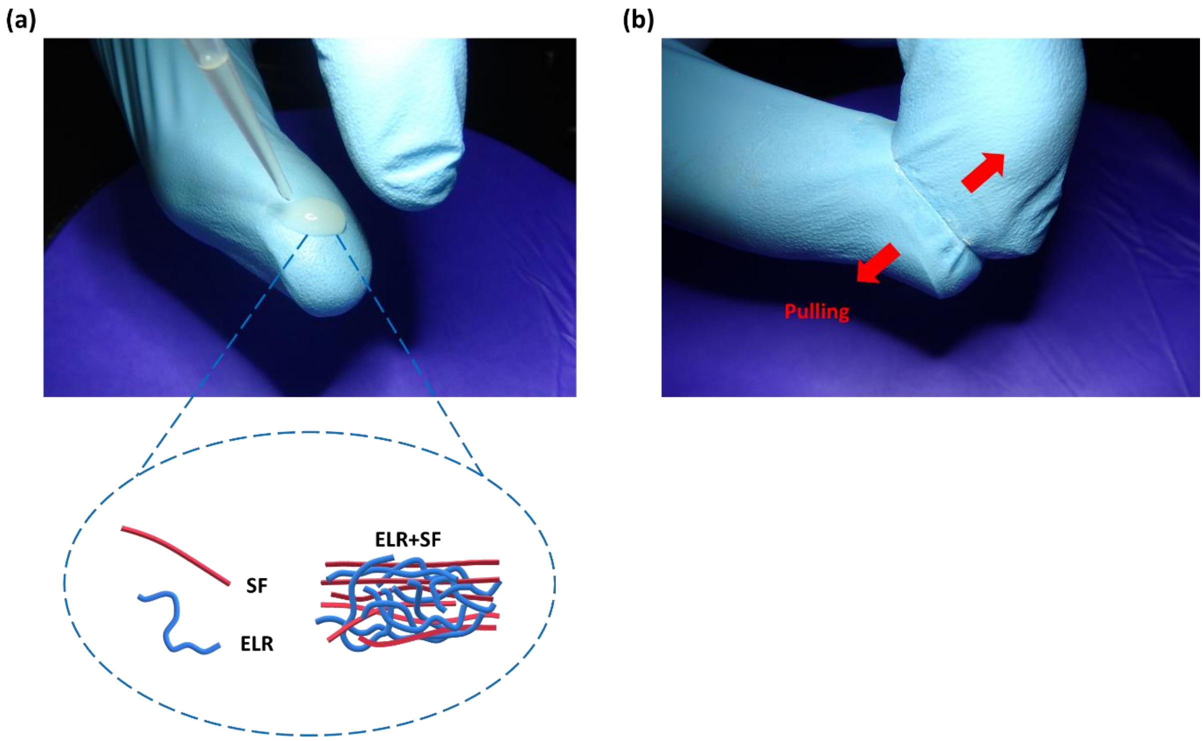

Figure S1. The appearance of ELR+SF bioadhesive. (a) Picture of ELR+SF bioadhesive deposited on the gloves. (b) Picture of the ELR+SF at the interface of the gloves while pulling.

Table S5. Comparison of adhesive formulations in similar studies.

Name of the adhesive	Composition	Tensile test	Animal tissue	Mechanical values	Curing time	Ref.
Composite bioadhesive	Elastin-like recombinamers and silk fibroin	Pull-off	Porcine skin	17.6 kPa	1 h	Present study
			Porcine bone	123.3 kPa	1 h	
SFT	Silk fibroin and tannic acid	Lap shear	Porcine skin	134.1 kPa	20 min	(Bai et al., 2019)
TASK	Silk fibroin and tannic acid	Lap shear	Rabbit skin	~10 kPa	20 min	(Gao et al., 2020)
SF@TA@HA	Silk fibroin, tannic acid and hydroxyapatite	Three-point bending	Porcine bone	922.8 kPa	35 min	(Bai et al., 2020)
(Ca)-modified SF	Calcium-modified silk fibroin	Peeling	Porcine skin	~400 N/m	24 h	(Seo et al., 2018)
TAPTRA	Tannic acid primed thermally-responsive polymers (gelatin, and poly (NIPAM-*co*-BA))	Pull-off	Porcine skin	~15 J/m^2 (gelatin), ~200 J/m^2 (poly (NIPAM-*co*-BA))	X	(Li et al., 2020)
Cat-ELP	Catechol-modified elastin-like polypeptides	Pull-off and lap shear	Porcine skin	37 kPa (pull-off) and 39 kPa (lap shear)	12-13 h	(Desai et al., 2020)
YKV and mYKV	DOPA-modified elastin-like polypeptides combined with crosslinkers (iron(III) nitrate, sodium periodate and tris(hydroxymethyl)phosphine)	Lap shear	Porcine skin	14.7 – 26.7 kPa	24 h	(Hollingshead et al., 2021)
SUP glue	Super charged elastin-like polypeptides and sodium dodecylbenzene sulfonate	Lap shear	Porcine skin	6 J/m^2	1 h	(Ma et al., 2021)
TAPE	Tannic acid, and poly(ethylene glycol)	Pull-off	Porcine skin	~180 kPa	1 min	(Kim et al., 2015)
PAAm-TA-KA	Polyacrylamide-tannic acid-kaolin hydrogels	Lap shear	Porcine skin	480 kPa	30 min	(Fan et al., 2020)
TNA hydrogel	Tannic acid and DNA hydrogel	Lifting	Rat subcutaneous tissue	~240 mg of tissue lifting	10 min	(M. Shin et al., 2015)
HA-CA hydrogel	Catechol-modified hyaluronic acid	Pull-off	Rat liver	1.4 kPa	10 min	(J. Shin et al., 2015)
GelMA-TA hydrogels	Gelatin methacrylate hydrogels and tannic acid	Lap shear	Porcine skin	81 kPa	1 min	(Liu et al., 2018)
Gel-TA	Gelatin crosslinked with tannic acid silver nitrate	Lap shear	Decellularized porcine small intestine submucosa	16.6 - 52.8 kPa	X	(Guo et al., 2018)

References:

Bai, S., Zhang, X., Cai, P., Huang, X., Huang, Y., Liu, R., Zhang, M., Song, J., Chen, X., & Yang, H. (2019). A silk-based sealant with tough adhesion for instant hemostasis of bleeding tissues. *Nanoscale Horizons*, *4*(6), 1333–1341. https://doi.org/10.1039/c9nh00317g

Bai, S., Zhang, X., Lv, X., Zhang, M., Huang, X., Shi, Y., Lu, C., Song, J., & Yang, H. (2020). Bioinspired Mineral–Organic Bone Adhesives for Stable Fracture Fixation and Accelerated Bone Regeneration. *Advanced Functional Materials*, *30*(5). https://doi.org/10.1002/adfm.201908381

Desai, M. S., Chen, M., Hong, F. H. J., Lee, J. H., Wu, Y., & Lee, S. W. (2020). Catechol-Functionalized Elastin-like Polypeptides as Tissue Adhesives. *Biomacromolecules*, *21*(7), 2938–2948. https://doi.org/10.1021/acs.biomac.0c00740

Fan, X., Wang, S., Fang, Y., Li, P., Zhou, W., Wang, Z., Chen, M., & Liu, H. (2020). Tough polyacrylamide-tannic acid-kaolin adhesive hydrogels for quick hemostatic application. *Materials Science and Engineering C*, *109*. https://doi.org/10.1016/j.msec.2020.110649

Gao, X., Dai, Q., Yao, L., Dong, H., Li, Q., & Cao, X. (2020). A medical adhesive used in a wet environment by blending tannic acid and silk fibroin. *Biomaterials Science*, *8*(9), 2694–2701. https://doi.org/10.1039/d0bm00322k

Guo, J., Sun, W., Kim, J. P., Lu, X., Li, Q., Lin, M., Mrowczynski, O., Rizk, E. B., Cheng, J., Qian, G., & Yang, J. (2018). Development of tannin-inspired antimicrobial bioadhesives. *Acta Biomaterialia*, *72*, 35–44. https://doi.org/10.1016/j.actbio.2018.03.008

Hollingshead, S., Torres, J. E., Wilker, J. J., & Liu, J. C. (2021). Effect of Cross-Linkers on Mussel- and Elastin-Inspired Adhesives on Physiological Substrates. *ACS Applied Bio Materials*. https://doi.org/10.1021/acsabm.1c01095

Kim, K., Shin, M., Koh, M. Y., Ryu, J. H., Lee, M. S., Hong, S., & Lee, H. (2015). TAPE: A medical adhesive inspired by a ubiquitous compound in plants. *Advanced Functional Materials*, *25*(16), 2402–2410. https://doi.org/10.1002/adfm.201500034

Li, B., Whalen, J. J., Humayun, M. S., & Thompson, M. E. (2020). Reversible Bioadhesives Using Tannic Acid Primed Thermally-Responsive Polymers. *Advanced Functional Materials*, *30*(5). https://doi.org/10.1002/adfm.201907478

Liu, B., Wang, Y., Miao, Y., Zhang, X., Fan, Z., Singh, G., Zhang, X., Xu, K., Li, B., Hu, Z., & Xing, M. (2018). Hydrogen bonds autonomously powered gelatin methacrylate hydrogels with super-elasticity, self-heal and underwater self-adhesion for sutureless skin and stomach surgery and E-skin. *Biomaterials*, *171*, 83–96. https://doi.org/10.1016/j.biomaterials.2018.04.023

Ma, C., Sun, J., Li, B., Feng, Y., Sun, Y., Xiang, L., Wu, B., Xiao, L., Liu, B., Petrovskii, V. S., Bin Liu, Zhang, J., Wang, Z., Li, H., Zhang, L., Li, J., Wang, F., Göstl, R., Potemkin, I. I., … Herrmann, A. (2021). Ultra-strong bio-glue from genetically engineered polypeptides. *Nature Communications*, *12*(1). https://doi.org/10.1038/s41467-021-23117-9

Seo, J. W., Kim, H., Kim, K. H., Choi, S. Q., & Lee, H. J. (2018). Calcium-Modified Silk as a Biocompatible and Strong Adhesive for Epidermal Electronics. *Advanced Functional Materials*, *28*(36). https://doi.org/10.1002/adfm.201800802

Shin, J., Lee, J. S., Lee, C., Park, H. J., Yang, K., Jin, Y., Ryu, J. H., Hong, K. S., Moon, S. H., Chung, H. M., Yang, H. S., Um, S. H., Oh, J. W., Kim, D. I., Lee, H., & Cho, S. W. (2015). Tissue Adhesive Catechol-Modified Hyaluronic Acid Hydrogel for Effective, Minimally Invasive Cell Therapy. *Advanced Functional Materials*, *25*(25), 3814–3824. https://doi.org/10.1002/adfm.201500006

Shin, M., Ryu, J. H., Park, J. P., Kim, K., Yang, J. W., & Lee, H. (2015). DNA/tannic acid hybrid gel exhibiting biodegradability, extensibility, tissue adhesiveness, and hemostatic ability. *Advanced Functional Materials*, *25*(8), 1270–1278. https://doi.org/10.1002/adfm.201403992
